# Mitigating Paxlovid™-induced drug‒drug interaction toxicity: an *in silico* insight

**DOI:** 10.3389/fphar.2024.1496630

**Published:** 2024-11-26

**Authors:** Giang H. Ta, Max K. Leong

**Affiliations:** Department of Chemistry, National Dong Hwa University, Hualien, Taiwan

**Keywords:** *in silico*, skin permeability, Paxlovid, nirmatrelvir, COVID-19

## Abstract

The clinical drug-drug interaction side-effects of Ritonavir, an ingredient in Paxlovid™, have been documented, highlighting the need to explore alternative administration methods for Nirmatrelvir, another drug in the Paxlovid™ combination. In this study, the skin permeability potential of Nirmatrelvir was assessed using various *in silico* models. The prediction results suggest that Nirmatrelvir could be administrated via the transdermal route, offering a promising avenue to enhance the efficacy of anti-COVID-19 agents.

## 1 Introduction

The first infected case of severe acute respiratory syndrome coronavirus 2 (SARS-CoV-2) was reported and identified in the city of Wuhan, China in December 2019. This initial case marked the onset of a swift outbreak that rapidly spread, leading to the infection of hundreds of million people and claimed millions of lives worldwide. This catastrophic health crisis has become the most severe pandemic in history, as documented by the World Health Organization (WHO) (https://covid19.who.int/). The associated disease caused by SARS-CoV-2 was officially designated as coronavirus disease 2019 (COVID-19) by WHO. The oral administration of Paxlovid™, which is a combination of Nirmatrelvir (PF-07321332) and Ritonavir (NM/r) developed by Pfizer, in the early stages of COVID-19 can reduce the severity of the disease. In fact, Paxlovid has been approved as the first-line small-molecule agent for the treatment of COVID-19 in outpatients (NHS England, 2022), including individuals affected by the Omicron variants. Notably, it is particularly recommended for patients aged 65 years and older. A recent study has demonstrated that the antiviral efficacy of Paxlovid against the latest omicron variants, namely, BA.286 and JN.1 (source: https://data.who.int/dashboards/covid19/variants), remains unchanged (Planas et al., 2024).

Nirmatrelvir was initially derived for the treatment of SARS by inhibiting the 3-chymotrypsin-like cysteine protease enzyme (3CL^pro^ or M^pro^), which plays a pivotal role in the viral cycle. Additionally, it has demonstrated potent interaction with the main SARS-CoV-2 protease M^pro^ and effectively prevent the virus replication (Fenton and Keam, 2022). Ritonavir, on the other hand, was originally developed as an HIV protease inhibitor. It also acts as a cytochrome P450 3A4 (CYP3A4) inhibitor and affects drug efflux, such as P-glycoprotein (P-gp). However, it has been observed that Nirmatrelvir can be metabolized by CYP3A4, which can lead to serious clinical side-effects in immunocompromised patients.

Transplant patients, for instance, often takes cyclosporine-A (CsA) as the first-line immunosuppressive agent to prevent graft rejection. Unfortunately, CsA is also metabolized by CYP3A4, and the inhibitory effect of Ritonavir on CYP3A4 can significantly and rapidly increase the plasma concentrations of CsA, leading to potentially fatal or life-threatening consequences (Fishbane et al., 2022). As such, the CsA dosing regimens for Paxlovid-treated patients should be cautiously monitored (Schuurmans and Hage, 2021). Furthermore, polypharmacy is common among elderly individuals (Khezrian et al., 2020). The administration of Ritonavir can lead to unfavorable interactions with other drugs, known as adverse drug-drug interactions (DDIs) (Ross et al., 2022). Therefore, it is of critical importance for clinicians to consult reliable resources such as https://covid19-druginteractions.org/, to ensure the safe and effective use of these medications, especially in patients with complex medication regimens and potential drug interactions.

It has been estimated that 7%–8% of patients treated with Paxlovid have experienced Paxlovid rebound, which is characterized by the reemergence of COVID-19 symptoms or a positive viral test results after previously testing negative (Birabaharan and Martin, 2022). The observed rebound in cases among Paxlovid-treated patients, occurring at a higher rate compared to untreated counterparts (Cohen and Brown, 2023), is believed to stem from insufficient drug exposure rather than drug resistance or immune impairment against the virus (Carlin et al., 2022). Consequently, there has been a recommendation to extend the duration of the treatment regimen from the original 5 days to 7–10 days. In the event of Paxlovid rebound, it is suggested to administrate additional 3–5 days of treatment (Rao and Singh, 2022). Nevertheless, it is of importance to note that prolonging the duration of Paxlovid treatment may increase the risk of experiencing severe side-effects when used in combination with other medications. Careful consideration of these potential side-effects and individual patient circumstances is crucial when making treatment decisions.

Thus, it can be concluded that the combination of NM/r has indeed yielded various clinical challenges, leading to the strong urge to design novel small antiviral molecules that can be administrated through alternative routes other than oral administration (Fenton and Keam, 2022). By removing Ritonavir from Paxlovid, it is possible to substantially reduce or even eliminate those clinically observed side-effects associated with Paxlovid. Of various alternative administration routes, topical and transdermal drug delivery (TDD) seemingly appear to be attractive options due to their noninvasive nature and self-administration potential. In addition, TDD methods can extend the duration of drug release and target immunogenic Langerhans cells within the skin (Prausnitz and Langer, 2008). These routes offer a pharmacokinetically safer and potentially more effective alternative compared to the oral route (Wu et al., 2022). Nonetheless, it is of importance to note that transdermal drug delivery poses a challenge, as only a limited number of drugs can effectively cross the skin barrier (Yu et al., 2021).

Although various transdermal drug delivery (TDD) methods, namely, ethosomes, microemulsions, iontophoresis, and microneedles, have been developed to enhance transcutaneous permeation, most antiviral agents still struggle to penetrate the skin due to their physicochemical properties (Ita, 2017; Wang et al., 2021). Among these properties, molecular weight (MW) and log *P* are considered key factors and are widely used to predict the skin permeability of novel compounds (Chen et al., 2018). Specifically, MW should be less than 500 Da, and the log *P*-value should range between one and 3 (Finnin and Morgan, 1999). These parameters align with Nirmatrelvir’s properties (MW = 499.53; log *p* = 0.44). Moreover, several antiviral drugs have been successfully delivered via TDD, such as indinavir (MW = 613.79; log *p* = 2.81), zidovudine (MW = 267.24; log *p* = −0.3), and acyclovir (MW = 225.21; log *p* = −1.0), despite having only one of these favorable physicochemical properties (Ita, 2017) (The antiviral drug MW and log *P* values were retrieved from DrugBank, accessed on 18 October 2024). This suggests that Nirmatrelvir could be a feasible candidate for transdermal delivery.

## 2 Materials and methods

The skin permeability of Nirmatrelvir was estimated by using various *in silico* approaches. This estimation was conducted using a previously published hierarchical support (HSVR), which is an innovative machine learning-based model (Wu et al., 2022), along with *SwissADME*, *pkCSM*, and *Skin Permeation Calculator* (SPC), which are available online (Table 1).

**TABLE 1:** Predicted log *K*_p_ values of Nirmatrelvir by *SwissADME*, *pkCSM*, *SPC*, and HSVR, converted log *P*_e_ values, skin permeability classification, and their references.

Predictor	log *K*_p_	log *P*_e_	Skin permeability	Reference
*SwissADME*	−7.81	−3.94	High	http://www.swissadme.ch/index.php
*pkCSM*	−2.71	−1.27	High	https://biosig.lab.uq.edu.au/pkcsm/prediction
SPC	N/A^a^	N/A	N/A	https://www.cdc.gov/niosh/topics/skin/skinpermcalc.html
HSVR	−0.34	−0.03	High	Wu et al. (2022)

^a^Not assessable.

## 3 Results and discussion

The predicted values of the logarithm of permeability coefficient or constant (*K*_p_) are listed in Table 1. Although log *K*_p_ values have been widely used to quantitatively assess skin permeability, the cut-off value for categorizing skin permeability levels has not yet to be clearly defined. However, Carrer et al. have defined skin permeability using the effective permeability coefficient (*P*_e_) classification as high, medium, and low if log *P*_e_ > − 6, − 8 < log *P*_e_ < − 6, and log *P*_e_ < − 8, respectively (Carrer et al., 2020). Accordingly, it is imperative to convert log K_p_ into log P_e_ in order to determine skin permeability of Nirmatrelvir. This was performed using the previously established conversion method between both metrics (Wu et al., 2022).

It is intriguing to observe that SPC failed to predict the log *K*_p_ value of Nirmatrelvir, whereas *SwissADME*, *pkCSM*, and HSVR displayed considerable variations in their predicted log *K*_p_ values. The failure of SPC can likely be attributed to its limited predictivity due to its narrower applicability domain, as evidenced by the fact that SPC could predict only *ca*. 80% of compounds in the previous study (Wu et al., 2022). All three predictors, nevertheless, unanimously identified Nirmatrelvir as highly skin permeable after converting log *K*_p_ into log *P*_e_ and applying skin permeability classification rules.

## 4 Conclusion

Thus, it can be concluded that the removal of Ritonavir from the combination of NM/r has the potential to reduce or even eliminate clinically adverse side-effects associated with the administration of Paxlovid. It is plausible to design the next-generation of anti-COVID-19 agent with minimal or no metabolism issues that, inevitably, can be time-consuming. Conversely, delivering Nirmatrelvir through topical or transdermal routes, such as a skin patch, can significantly enhance its clinical efficacy and pharmacokinetic safety while simultaneously addressing the issue of Paxlovid rebound. This approach offers a quick and cost-effective solution. However, further scientific research and clinical trials are required to support these conclusions and determine the effectiveness, safety, and feasibility of the proposed modifications. These steps are essential to ensure the successful development and implementation of such a strategy in the treatment of COVID-19.

## Data Availability

The original contributions presented in the study are included in the article/supplementary material, further inquiries can be directed to the corresponding author.
